# Some grand challenges in environmental chemistry

**DOI:** 10.3389/fchem.2013.00001

**Published:** 2013-02-26

**Authors:** Steven L. Suib

**Affiliations:** Unit 3060, Department of Chemistry, University of ConnecticutStorrs, CT, USA

## Overview

Environmental chemistry is a very broad topic (Jardim, 1998; Ventura et al., 2009; Kubicki and Mueller, 2010; Steinbach and Hoffmann, 2010; Subramaniam, 2010; Caumul, 2011; Gao et al., 2011; Humphris, 2011; Wirth et al., 2011). This subject spans aspects of all states of matter and various phenomena including pollution, (Ashmore and Nathanail, 2008; Busca, 2009; Carls and Meador, 2009; Feldmann et al., 2009; Steinbach and Hoffmann, 2010; Gao et al., 2011; Humphris, 2011; Wirth et al., 2011) the generation of new materials, health, medicine, and energy. With ever increasing population constraints on resources demand interest in sustainable technologies and these must be green in nature.

In terms of energy, there are environmental chemistry concerns regarding solar, geothermal, nuclear, fossil fuel, and other forms of energy. In the area of materials, nano-size systems are currently of tremendous interest and such systems pose environmental and health threats that are just beginning to be formulated (Ashmore and Nathanail, 2008; Gao et al., 2011; Humphris, 2011; Wirth et al., 2011). Feedstocks of all types are subject to contamination from a variety of sources. Analyzing such feedstocks and determining exact concentrations can be a challenge. Computational modeling studies are often useful in understanding such complex systems (Ventura et al., 2009; Kubicki and Mueller, 2010). Continual new regulations and protocols based on new data influence our world every day.

The characterization of materials is an area that continues to improve. The chemical and physical properties of systems and their environmental effects are often challenging especially with real world samples that are not pristine and may have a multitude of components. These days composite materials or multi-component systems are being developed to solve complex problems. The synergetic effects of such composites are often not well known and the potential environmental problems associated with such composites are also not well known (He et al., 2008; Carls and Meador, 2009). The development of novel methods of analysis to study such complex systems and concomitant environmental chemistry problems is a major area of research. *In situ* monitoring and miniaturization of such instrumentation are some of the goals of this area.

There is a continual need for basic studies of air, water, soil, and artificial/man-made materials. How all of these systems interact with various life forms is of considerable interest. Interactions of bacteria, viruses, and cells in these different environments continue to be developed. The political, legal, and social aspects of results of these studies are constantly being explored.

## Innovation

There need to be new developments in the accurate measurements of environmental threats. This includes exact procedures to be used that are acceptable to everyone. New instrumentation that allows *in situ* monitoring of environmental chemistry would be invaluable. Such real time monitoring should help elucidate reaction mechanisms and provide ideas about how to combat environmental problems. Methods to extract natural chemicals from the environment to use in remediation and the generation of materials would be extremely helpful as regards sustainability and resource management.

## Future challenges

Translating basic research into applied areas will be the subject of several studies in the future. Reclamation of land from contaminated landfills, regeneration of polluted sources of water and air, and use of wastes need to be pursued. Catalysis and rational design of materials to be used in such processes will be key factors in realizing these goals (Subramaniam, 2010). How to measure properties of living systems in real time including imaging is a grand challenge that has multiple implications in the field of environmental chemistry. Integration of new materials, processes, protocols, and analyses to understand environmental chemical systems is another important grand challenge. Novel intelligent sensors also need to be developed to monitor environmental species of all types.

## Conclusions

The area of environmental chemistry is ripe for continued growth and development. As populations continue to grow and there are more and more demands on all resources there will be an intense need for green materials, green energy sources, green processes, a green viable solutions to problems that need to be solved to promote growth in a healthy environment.
